# Comparison of Typical and Atypical Community Acquired Pneumonia Cases in Hospitalized Patients in Two Tertiary Centers in Riyadh, Saudi Arabia

**DOI:** 10.3390/arm93060058

**Published:** 2025-12-13

**Authors:** Abdullah Almufleh, Abdulrahman Altuwayjiri, Abdulmalik Alshehri, Abdulaziz Alzouman, Abdulhadi Alotaibi, Abdulrahman Alsaedy

**Affiliations:** 1 College of Medicine, King Saud bin Abdulaziz University for Health Sciences, Riyadh 14611, Saudi Arabia; almufleh404@ksau-hs.edu.sa (A.A.); altuwayjiri388@ksau-hs.edu.sa (A.A.); alshehri0405@ksau-hs.edu.sa (A.A.); alzouman394@ksau-hs.edu.sa (A.A.); alotaibi406@ksau-hs.edu.sa (A.A.); 2 Department of Medicine, Division of Infectious Diseases, King Abdulaziz Medical City, Ministry of the National Guard Health Affairs, Riyadh 14611, Saudi Arabia; 3 King Abdullah International Medical Research Center, Riyadh 14611, Saudi Arabia

**Keywords:** community-acquired pneumonia, atypical pneumonia, *Mycoplasma pneumoniae*, *Chlamydia pneumoniae*, *Legionella pneumophila*, molecular testing, multiplex PCR, epidemiology, Saudi Arabia

## Abstract

**Highlights:**

**What are the main findings?**

- Of all episodes hospitalized with CAP, 65% underwent molecular testing via PCR.
- Of those, atypical pneumonia organisms were identified in 2.09%, with *Mycoplasma pneumoniae* being the most common identified atypical pathogen.

**What is the implication of the main finding?**

- Molecular testing should be utilized more frequently in CAP hospitalized patients.

**Abstract:**

Background/Objectives: Community-acquired pneumonia (CAP) is classified into typical and atypical forms, with *Mycoplasma pneumoniae*, *Chlamydia pneumoniae*, and *Legionella pneumophila* being the most common atypical pathogens and *Streptococcus pneumoniae* and *Haemophilus influenzae* the most common typical organisms. This study aimed to compare the prevalence, demographics, and clinical outcomes of hospitalized typical and atypical CAP patients. Methods: A cross-sectional study was conducted from January 2016 to June 2022 at two tertiary hospitals in Riyadh, Saudi Arabia. All inpatients diagnosed with CAP by imaging and clinical findings were included, excluding viral cases. Outcomes measured included pathogen testing and identification, hospitalization duration, ICU stay, and in-hospital mortality. Results: Among 1238 CAP hospitalizations, 65% underwent molecular testing, with atypical pathogens detected in 17 cases (2.09%). *Mycoplasma pneumoniae* was the most common organism. The cases had an almost equal male-to-female ratio. Mean hospitalization was 12 days overall versus 4 days for atypical pneumonia. Of 265 ICU admissions, none tested positive for atypical CAP. Overall mortality was 6.94%, with no deaths in atypical pneumonia positive patients. Conclusions: PCR molecular testing was performed in 65% of patients hospitalized with CAP, and atypical pneumonia organisms were uncommon in these patients, with *Mycoplasma pneumoniae* being the most common. Clinical outcomes were more favorable for these patients. Expanding molecular testing may improve pathogen detection and guide target management.

## 1. Introduction

Annually, 2.7 million people worldwide die of pneumonia [1]. It is one of the most common causes of death globally [1]. Pneumonia is defined as an infection of the lower respiratory tract. Based on the setting of transmission, it is divided into community-acquired and hospital-acquired pneumonia. Community-acquired pneumonia, “CAP”, can be defined as the acquisition of pneumonia infection outside health care settings. CAP can then be divided into typical and atypical CAP. Typical and atypical CAP are different in the causative agents, clinical features, imaging findings, disease course, and treatment coverage [2]. The common organisms causing typical pneumonia include *Streptococcus pneumoniae* and *Haemophilus influenzae* [3]. This type of pneumonia usually presents with fever, productive cough, and pleuritic chest pain [4]. On imaging, typical pneumonia usually presents as lobar opacity [5]. On the other hand, the most common atypical pneumonia organisms include *Mycoplasma pneumoniae*, *Chlamydia pneumoniae*, and *Legionella pneumophila* [2]. Additionally, the mentioned pathogens as well as Bordetella pertussis and Bordetella parapertussis can cause non-pneumonia lower respiratory tract infection [6]. The prevalence of atypical pneumonia is known to be less than that of typical pneumonia with a much higher prevalence in outpatient settings [2]. While Streptococcus pneumonia is the most common cause of pneumonia among all age groups, atypical pathogens commonly affect children and young adults as seen in many studies [2,7]. Atypical pathogens are more frequently linked to extrapulmonary manifestations, and while their course typically tends to be milder than that of typical pneumonia, *Legionella pneumophila* infections remain an exception, as they can cause severe and fatal form of pneumonia [2,8,9]. Common symptoms of atypical pneumonia are low-grade fever, headache, earache, and sore throat [2]. Finally, since the causative agents of atypical pneumonia are different from those of typical pneumonia, the treatment coverage is different, and the use of antibiotics in an optimal setting should be aimed against the specific organisms. Hence, it is important to identify and specifically target the causative agents. However, unfortunately, the knowledge of atypical pneumonia particularly is still insufficient.

Though it is theoretically different, the distinction of pneumonia caused by atypical agents from other pneumonia infections is still impossible through clinical presentation alone [2]. In addition, the identification of these organisms through performing culture is usually not conducted, as it is difficult and requires expertise [2]. Therefore, while there is no established gold standard for detection of atypical pneumonia, it is usually performed through PCR, direct antigen detection and serology [2,3]. In many cases in real practice, however, after the diagnosis of CAP, patients do not undergo further investigations to identify the specific causative agents [2]. This is likely due to the use of empirical antibiotics that diminish the need to specify the bacteria, but that is changing owing to the increasing awareness of the significance of the rational use of antibiotics [2]. It is now recommended to conduct diagnostic tests in all CAP hospitalized patients [10]. The commonly used B-lactam antibiotics are ineffective against atypical pathogens such as *Mycoplasma pneumoniae*, highlighting the importance of accurate pathogen identification [11]. Remarkably, a recent retrospective study performed in Peking suggested that the incidence of Mycoplasma pneumoniae infection was even higher than that of Streptococcus although it was on outpatients [12]. In addition, the incidence of atypical pneumonia might increase as a result of aging populations, the usage of immunosuppressive drugs and the emergence of antibiotic-resistant organisms [13]. All these factors indicate the significance of the distinction between these types of pneumonia, but this does not happen often in practice.

This paper aims to compare the prevalence, demographics, and clinical outcomes of hospitalized patients with typical versus atypical CAP in King Abdulaziz Medical City (KAMC) and King Abdullah Specialized Children’s Hospital (KASCH), Riyadh, Saudi Arabia.

## 2. Materials and Methods

### 2.1. Study Design and Setting

This study was an observational cross-sectional study that aimed to compare the prevalence, demographics, and clinical outcomes of typical and atypical CAP inpatients seen at two of the largest tertiary care centers in the Middle East: King Abdulaziz Medical City (KAMC) and King Abdullah Specialized Children’s Hospital (KASCH), Riyadh, Saudi Arabia.

### 2.2. Data Collection

Patients meeting the inclusion criteria mentioned below are included in this study, and their paper and electronic charts and medical records were reviewed. Microsoft Excel was used to collect data and the JMP standard edition 114 program to analyze the data. The population of this study was all inpatients diagnosed with community-acquired pneumonia using clinical and radiological findings during the period between 2016 and 2022. The exclusion criteria were all patients with viral infective agents. The identification of pathological agents was performed through a standard laboratory-validated PCR multiplex only. PCR multiplex testing was not systematically offered to all hospitalized CAP patients. However, testing was a decision based on the treating physician’s judgment. The subjects’ data were extracted from medical records by King Abdullah International Medical Research Center.

### 2.3. Statistics Presentation

As for the presentation of statistics, frequencies and percentages were used to describe categorical data such as sex and type of infective agent; mean and standard deviation were used to present numerical data such as height, weight, and duration of ICU stay.

### 2.4. Tools Used

Three different 2.0 PCR multiplex panels were used in these two tertiary centers during the course of this study. From 2016 to late 2017, a panel that included *Mycoplasma pneumonia*, *chlamydia pneumoniae*, *Bordetella pertussis*, and *Bordetella parapertusis* was used along with a separate PCR assay for *Legionella pneumophila*. From late 2017 until May 2020, a different PCR kit was implemented that targeted only *Mycoplasma pneumonia* and *chlamydia pneumoniae*. Beginning in May 2020 and continuing through 2022, the first PCR multiplex was again adopted without the addition of the separate *Legionella pneumophila* assay.

## 3. Results

### 3.1. Prevalence of Atypical CAP

A total of 1238 hospitalized episodes diagnosed with CAP were included. All these episodes represent 1022 patients. Of the 1238 episodes diagnosed with CAP, 811 episodes (65.5%) had undergone PCR multiplex testing for at least one atypical organism, and then 17 episodes were found positive for an atypical pneumonia pathogen. This indicates a 2.09% prevalence of atypical pneumonia among the tested episodes (95% CI: 1.1–3.1%), while 427 cases (34%) were not subjected to pathogen-specific testing.

### 3.2. Demographics

**Figure 1:**
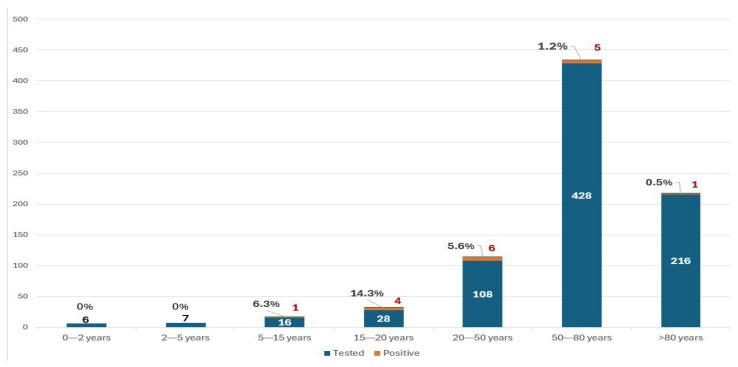
Number of CAP cases in each age group who underwent molecular testing for atypical pathogens, the number of cases that were found positive (in red), and the percentage of atypical pathogens positivity.

The percentages of male (49.23%) and female (50.77%) patients were almost equal. The age distribution was skewed, with the mean age being 66 years and the median age being 72 years. As seen in (Table 1), the age group with highest number of CAP episodes in general was 50–80 years. The oldest patient included was a patient aged 114 years. The number of cases that underwent PCR testing and the number of cases that was found positive across all age groups are shown in (Figure 1). Atypical pathogens were detected most frequently in patients aged 20–50 years, even though the number of tested patients aged 50–80 years was nearly fourfold higher. However, after accounting for the size of each tested age group, the 15–20 age group demonstrated the highest proportion of atypical pathogens positivity as can also be seen in (Figure 1).

### 3.3. Pathogens

Among atypical pneumonia episodes, the most commonly identified microorganism was *Mycoplasma pneumoniae*, though all of those tested for it were also tested for Chlamydophila pneumoniae as observed in Table 2. The difference in organisms tested is attributed to the use of three different PCR multiplex panels through the course of this study, as mentioned previously.

### 3.4. Hospitalization and ICU Stay

The average number of days of hospitalization for all included cases was 12 days. However, the average number of days of hospitalization of atypical pneumonia patients was 4 days. Of all episodes, 265 were admitted to ICU, none of whom were atypical pneumonia positive. Among ICU admitted patients, 55% were of the age group 50–80 years. For all ICU-admitted patients, the median ICU stay was found to be 9 days; the median was chosen for calculation because one case stayed 535 days in ICU, affecting the mean.

### 3.5. Outcome

All atypical pneumonia patients were discharged alive. On the other hand, seventy-nine of typical CAP patients died before discharge making the mortality rate 6.94%. Of these, 50% were between 50 and 80 years old, and 44% were older than the age of 80 years.

### 3.6. Recurrence

The number of patients with recurrent disease and recurrent hospitalization was 207, and that represents almost 20% of all patients. Recurrence was defined as a second admission of pneumonia more than 30 days following the first infection. All cases of recurrence were either not tested or tested negative for atypical pathogens. Furthermore, all the 17 patients who had been confirmed to have atypical pneumonia did not present again with pneumonia. Most recurrences (53%) were in the age group between 50 and 80 years. The average number of recurrences in patients who had a recurrence was almost 2 times “~2.4”. An elderly female and an adolescent female had the highest number of recurrences with six episodes each.

### 3.7. Readmission

The number of cases readmitted within the first 30 days following the latest discharge was 72, which represents 5.8% of all cases. Five cases were readmitted twice, and two were admitted three times. A second readmission was defined as a readmission occurring within 30 days of discharge from the first readmission. Two patients had been readmitted twice in two different episodes.

### 3.8. Incidence per Year

**Figure 2:**
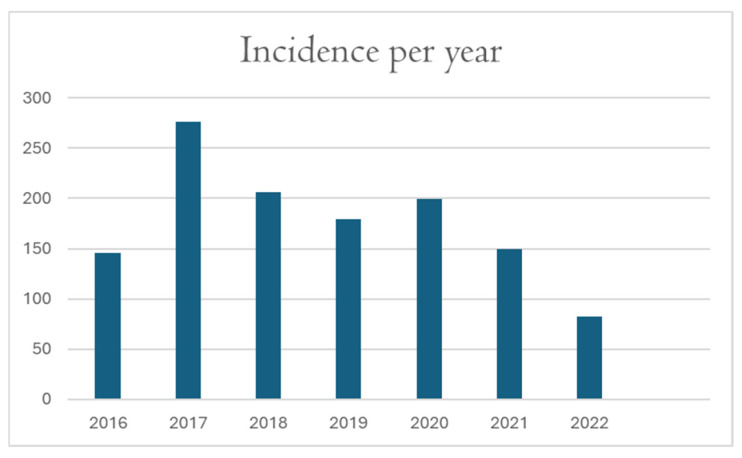
Annual Hospitalizations for Community-Acquired Pneumonia (2016–2022).

Regarding the year of presentation, the year found to have the highest number of hospitalizations for CAP was 2017 as shown in (Figure 2). Findings were similar for patients with atypical pneumonia, as 2017 was the year with the highest number of diagnoses of atypical pneumonia. However, the only difference was that no further positive identifications of atypical pneumonia pathogens were made beyond May 2020 despite the continued and extensive PCR testing since 2020 and afterwards.

## 4. Discussion

The prevalence of atypical pneumonia found in this study was low compared to other studies in Saudi Arabia and around the world, such as a retrospective study performed in the Al Qassim region in 1992 on patients of both types of CAP, which suggested that most of the patients with CAP were diagnosed with either Streptococcal infection (26%) or *Mycoplasma pneumonia* (24%) [14]. Additionally, a recent study in a single center in Asir, Saudi Arabia on all identified causes of pneumonia found that only 6% were caused by atypical pathogens [15]. Specifically, *Legionella Pneumophila* was the only identified atypical organism [15]. Nevertheless, the prevalence found in our study was slightly similar to most previous studies performed exclusively on hospitalized patients. For instance, a secondary analysis worldwide performed exclusively on inpatients revealed a prevalence of atypical pathogens of 4.6% [16]. In Spain, however, it was different, as the prevalence was found to be 2%, which is the same as the prevalence found in our study [16]. Nevertheless, a prospective analysis of four Dutch inpatient cohorts reported a 20% prevalence of atypical pathogens, likely reflecting the study’s extensive use of microbiological testing [7].

These low numbers might be attributed to the lack of molecular testing for all patients as was found in our study. Alternatively, it could also be attributed to the lack of implementation of serology tests, relying exclusively on PCR multiplex, or the usage of empirical antibiotics. For example, a study comparing the results of PCR and serology suggested that the use of both serology and PCR is the ideal way to identify atypical pathogens [17]. A recent study in Tehran involving outpatients with clinical features of atypical pneumonia found that PCR testing provided superior sensitivity and rapidity in detection of *Mycoplasma pneumonia* compared to culture testing [18].

The low detection rate of atypical pathogens in our cohort is also likely to be related to the selective use of molecular testing, which was mainly performed in clinically sicker patients. Although no formal institutional guidelines exist to determine which patients undergo PCR testing, several treating physicians reported that testing is typically ordered for cases with severe presentation or poor response to initial empiric therapy. This can be observed in our data in that the average length of hospitalization among patients who underwent molecular testing was 14 days, compared with only 7 days among those who were not tested. Testing patients with milder disease could have resulted in a significantly higher positive rate of atypical pneumonia.

The relatively low prevalence in our study may otherwise be explained by the limited testing of *Legionella Pneumophila*, a common and well-recognized cause of atypical pneumonia. A recent meta-analysis on 219 observational studies suggested a 4.6% global mean proportion of *Legionella* as the causative agent for CAP, and the rates appear highest in high-income countries [19]. Another earlier meta-analysis of fifty studies found an overall prevalence of 2.7% for *Legionella Pneumophila* [20]. All in all, the limited testing of *Legionella Pneumophila* in our centers could have affected the accuracy of our reported prevalence.

The other findings in our paper, such as the peak age of both typical and atypical pneumonia, were consistent with many studies, such as a meta-analysis conducted in 2015 [7]. The percentage of readmission within 30 days was similar to that of a single-center study conducted in Japan, which found the rate to be 5.5%, though it focused on symptom recurrence rather than rehospitalization [21].

It was noted in our study that since 2020 and the introduction of the COVID-19 pandemic, the rate of cases undergoing tests to identify pathogens significantly increased from only 57% pre-COVID-19 to 82% post-COVID-19. Nevertheless, no single case was positive for atypical organisms since 2020. This finding is similar to a Chinese retrospective study that compared the detection rate of pneumonia organisms in tested children patients in 2019 and 2020, which found that the detection rate of *Mycoplasma pneumonia* dropped significantly from 48% in 2019 to 16% in 2020 [22]. Furthermore, another Chinese study comparing the detection rate of CAP pathogens in tested children in 2019, 2020, and 2023 also found that the rate of detection of Mycoplasma pneumonia in 2020 at 9% was the lowest compared to 23% in 2019 and 35% in 2023 [23].

The rate of testing for atypical organisms found in our paper (65%) was much higher than that of Europe, which was found in the same secondary analysis mentioned above to be 46% [9].

The overall CAP in-hospital mortality, which was in our study 6.94%, is similar to the 6% found in the related study performed in Al-Qassim, Saudi Arabia mentioned earlier [14]. However, this is significantly lower than the 21% in a recent retrospective study in Pakistan [24]. It was also lower than the 18% reported in a German meta-analysis of in-hospital mortality in CAP patients [25]. However, our in-hospital mortality was similar to a retrospective study conducted over 20 years in Hospital Clinic, Barcelona and found a rate of 8% [26].

Limitations of this study include that it was conducted only in one tertiary center, the primary reason for hospitalization was not investigated, the lack of standardized guidelines to guide the decision of choosing patients for molecular testing, the absence of culture confirmation, and the lack of serology testing. Another limitation is that the cases that underwent molecular testing are possibly the cases with more severe clinical presentation, and this probably affected the prevalence of atypical organisms.

## 5. Conclusions

This study showed that molecular testing was performed only in 65% of hospitalized CAP patients. Of those, atypical pneumonia organisms were identified in 2.09%, and *Mycoplasma Pneumonia* was the most identified atypical organism. Recurrence, readmission, length of hospitalization, ICU admissions, and in-hospital mortality were all more favorable in patients with a confirmed atypical bacterium.

## Figures and Tables

**Table 1:** Age distribution of all hospitalized CAP cases included in the study.

Age Group	(n)
0–2 years	9
2–5 years	10
5–15 years	21
15–20 years	41
20–50 years	135
50–80 years	676
More than 80 years	346
**Total**	**1238**

**Table 2:** Microorganisms Identified in Atypical Pneumonia Cases.

Organism	n. of Cases Tested	Positive Cases (n)	Percentage (%)
Mycoplasma pneumoniae	808	14	1.73%
Chlamydophila pneumoniae	808	3	0.37%
Bordetella parapertussis	392	0	0
Bordetella pertussis	390	0	0
Legionella pneumophila	180	0	0

## Data Availability

The data presented in this study are available on request from the corresponding author. The data are not publicly available due ethical restrictions as it is patients’ raw data.

## Abbreviations

The following abbreviations are used in this manuscript:

CAP	Community-Acquired Pneumonia
ICU	Intensive Care Unit
PCR	Polymerase Chain Reaction
KAMC	King Abdulaziz Medical City
KASCH	King Abdullah Specialized Children Hospital
KAIMRC	King Abdullah International Medical Research Center
